# Ovine Forestomach Matrix as a Substrate for Single-Stage Split-Thickness Graft Reconstruction

**Published:** 2013-11-07

**Authors:** Jeremy Simcock, Barnaby C. H. May

**Affiliations:** ^a^University of Otago Christchurch, Christchurch, New Zealand; ^b^Mesynthes Limited, Lower Hutt, New Zealand

**Keywords:** biomaterial, carcinoma, ovine forestomach matrix, reconstruction, split-thickness graft

## Abstract

**Objective:** Split skin graft reconstruction of scalp defects often leaves an obvious contour defect. Here, we aimed to demonstrate the use of a decellularized extracellular matrix biomaterial, termed ovine forestomach matrix (OFM), as a substrate for split-thickness skin grafts (STSGs) for scalp reconstruction. **Methods:** Following full-thickness tumor excision, OFM was applied directly to skull periosteum, and then an STSG was applied. Participants were monitored for graft take, epithelialization, and cosmetic outcomes. **Results:** Participants responded well to the procedure with more than 95% graft take in 4 participants, and 100% epithelialization of the grafts after 2 weeks. A 30% graft take was observed in the fifth participant due to local infection and partial necrosis of the graft. Ovine forestomach matrix was remodelled with time and the regenerated dermis was well vascularized and had robust and ordered collagen deposition. **Conclusions:** This series demonstrates that OFM can serve as a temporary dermal scaffold to support an overlying STSG and allow for a single-stage grafting procedure.

Reconstruction of skin defects may be performed by skin grafting procedures. Full-thickness skin grafts result in a more durable reconstruction due to the larger proportion of dermis placed into the defect than split-thickness skin grafts (STSGs). Because of limited full-thickness skin graft donor sites, STSGs are used in larger defects. Two-stage grafting procedures have been developed whereby a dermal substitute is grafted into the defect under an artificial epidermis, which is subsequently replaced by an STSG. There is a clinical need to replace the relative complexity of 2-stage grafting procedures with robust single-stage procedures without compromising clinical outcomes. However, the feasibility and success of single-stage procedures is dependent on the vascularity of the underlying tissue. To overcome these limitations, collagen-based dermal substitutes have been investigated as temporary substrates for an overlying STSG. This approach creates a composite graft, whereby the underlying dermal substitute is rapidly vascularized and therefore can support epithelial proliferation of the STSG, leading to closure of the defect and dermal regeneration. The dermal substitute, human acellular dermal matrix (eg, Alloderm) has been investigated for STSG composite grafting in the treatment of burns,^1^^-^3 traumatic skin loss,^2^^,^^4^^,^^5^ and tumor excision.^6^^-^8

Ovine forestomach matrix (OFM) is a decellularized extracellular matrix biomaterial developed for wound healing and tissue regeneration applications and is cleared by the US Food and Drug Administration for dermal indications. Ovine forestomach matrix comprises mainly collagens I and III arranged as native fibres that retain the 3-dimensional architecture seen in tissue ECM.^9^ Additional structural (eg, collagen IV, fibronectin, and elastin), signalling (eg, glycosaminoglycans and heparin sulphate), and adhesion molecules (eg, laminin) are also present. Ovine forestomach matrix is nonantigenic, and it undergoes cellular infiltration and subsequent remodelling leading to regeneration of missing or damaged tissues. In preclinical models, OFM has been shown to be angioinductive and is rapidly revascularized,^10^ and in clinical studies, OFM treatment resulted in well vascularized granulation tissue in chronic venous ulcers.^11^ These previous findings suggested that OFM may be suitable for composite grafting with STSGs, where clinical success is reliant on the ability for the substrate to rapidly revascularized and provide the requisite nutrients and immune components to the overlying STSG.

## METHODS

### Case studies

**Figure 1:**
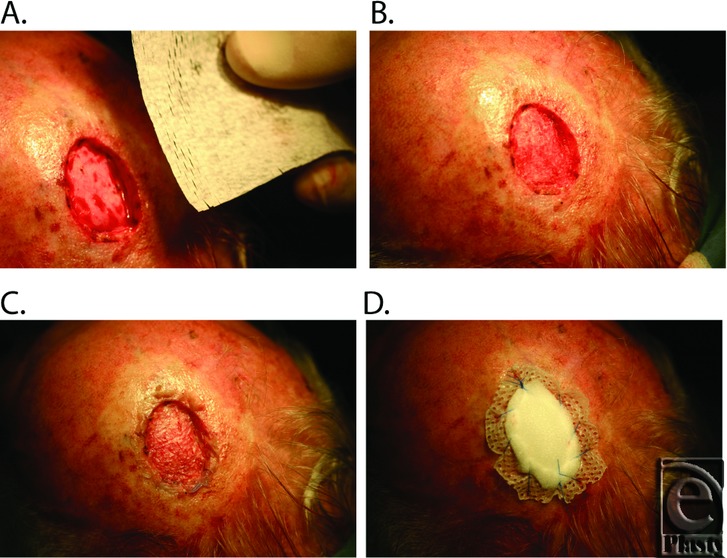
Representative images of the tumor resection and single-stage split-thickness grafting. (*a*) Excisional defect following tumor excision and meshed OFM prior to rehydration. (*b*) Rehydrated OFM cut to size and placed within the defect to conform to the underlying periosteum. (*c*) Meshed STSG in contact with the underlying OFM. (*d*) Secondary dressings secured to the perimeter of the excision.

The case series was approved by an institutional review board (Upper South A Regional Ethics Committee, New Zealand) and registered with the Australian New Zealand Clinical Trials Registry (http://www.anzctr.org.au/). Five participants were selected on the basis of the inclusion and exclusion criteria listed in Table 1 and all tumors were confirmed by pathology prior to the procedure. The procedure was conducted under either local or general anesthetic. A full-thickness excision down to but not including the pericranium was used to remove the tumor and a 5- to 10-mm margin (Fig 1a). Ovine forestomach matrix (Endoform, Mesynthes Limited, New Zealand) was meshed by either hand or a skin graft mesher at a ratio of 1.5:1 (Zimmer) and then trimmed to fit the excisional defect. The material was rehydrated in sterile saline for a minimum of 5 minutes and placed into the defect to contact the underlying periosteum (Fig 1b). An STSG (approximately 0.25-mm thick) was harvested from the thigh of each participant, using either a dermatome (Zimmer Machinery Corporation, Cowpens, South Carolina) or a hand knife. The graft was meshed by hand, cut to fit the defect, and then placed over the OFM, making sure the OFM and STSG were in contact (Fig 1c). A nonadherent dressing (Mepitel, Mölnlycke Health Care, Sweden) was placed over the graft, then a bolster of foam was sutured in place to ensure close contact between the STSG, OFM, and underlying periosteum (Fig 1d). The secondary dressing was removed 7 days following surgery and the graft imaged and evaluated for percentage graft take and epithelialization, based on the total area of the defect. A silver-based hydrogel (Silvasorb; Medline Industries, Inc, Mundelein, Illinois) was used to treat any suspected bacterial infection. The defect was re-dressed using a nonadherent dressing, as required, and reevaluated weekly for the first fortnight, then monthly or as required. At final review, the healed wounds were assessed for contour defect and scalp mobility by palpation.

### Histology and immunohistochemistry

Excised tissues were fixed with 4% formalin, paraffin embedded and stained. Gomoris’ Trichome staining was conducted as previously described.^10^ Anti-CD34 immunohistochemistry was conducted as previously described^10^ using a mouse antihuman CD34 (Abcam Plc, Cambridge, England) monoclonal antibody. Slides were imaged using a CX-31 microscope (Olympus Imaging America Inc, Center Valley, Pennsylvania) fitted with a DP12 digital camera (Olympus).

## RESULTS

**Figure 2:**
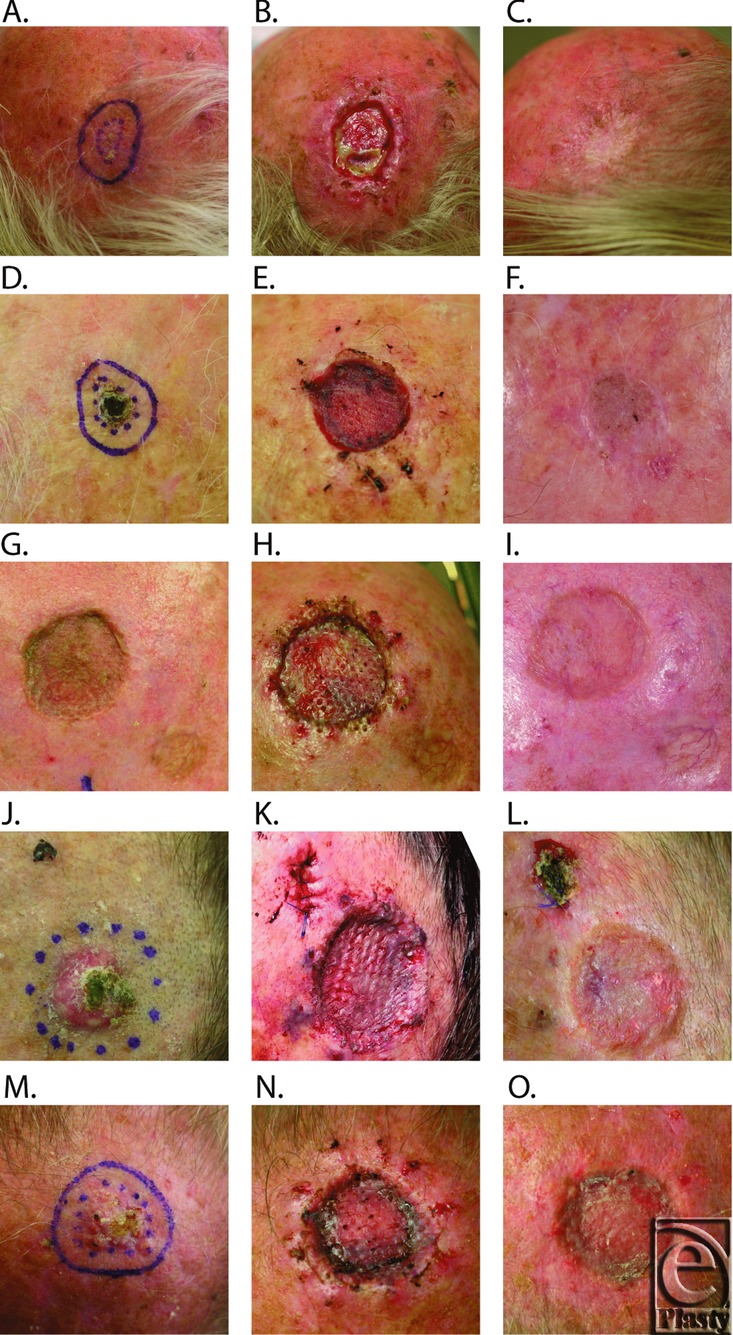
Representative images of the study participants B001 (2.A., 2.B., 2.C.), B002 (2.D., 2.E., 2.F.), B003 (2.G., 2.H., 2.I.), B004 (2.J., 2.K., 2.L.), and B005 (2.M., 2.N., 2.O.), prior to tumor excision (2.A., 2.D., 2.G., 2.J., 2.M.) and 1 week following surgery (2.B., 2.E., 2.H., 2.K., 2.N.). Surgical site following healing; 2.C., 40 weeks; 2.E., 16 weeks; 2.I., 16 weeks; 2.L., 4 weeks (prior to reexcision); 2.O., 4 weeks (prior to reexcision).

Participants (B001 through B005) enrolled in the study were all male, 61 to 83 years old, presenting with either an squamous cell carcinoma (SCC) (n = 4) or basal-cell carcinoma (BCC) (n = 1), located on the scalp (Table 2). The tumor size, estimated at enrolment, ranged from 1.2 to 4.6 cm^2^, and tumors had been present for approximately 2.5 to 9 months. Following tumor excision, the full-thickness wounds were approximately 5 to 10 cm^2^. Ovine forestomach matrix could be meshed using a surgical skin graft mesher and once rehydrated was easy to handle and conformed well to the underlying periosteum. One week postsurgery, 4 of the participants had more than 95% graft take (B002, B003, B004, and B005), while the fifth participant, B001, had a 30% graft take. The low graft take in participant B001 resulted from a local infection and partial necrosis of the graft (Fig 2b), which was managed with a silver-containing hydrogel. Complete epithelization of all grafts occurred in 2 weeks, except for participant B001 where infection delayed complete epithelialization to 8 weeks.

Participants B001, B002, and B003 were available for long-term follow-up (Fig 2). The epithelium remained stable throughout follow-up (minimum follow-up of 6 months, range 7-9 months). Regenerated dermal tissues were well vascularized, elastic, and mobile over the underlying periosteum. Contour defects were judged to be mild via subjective observation.

**Figure 3:**
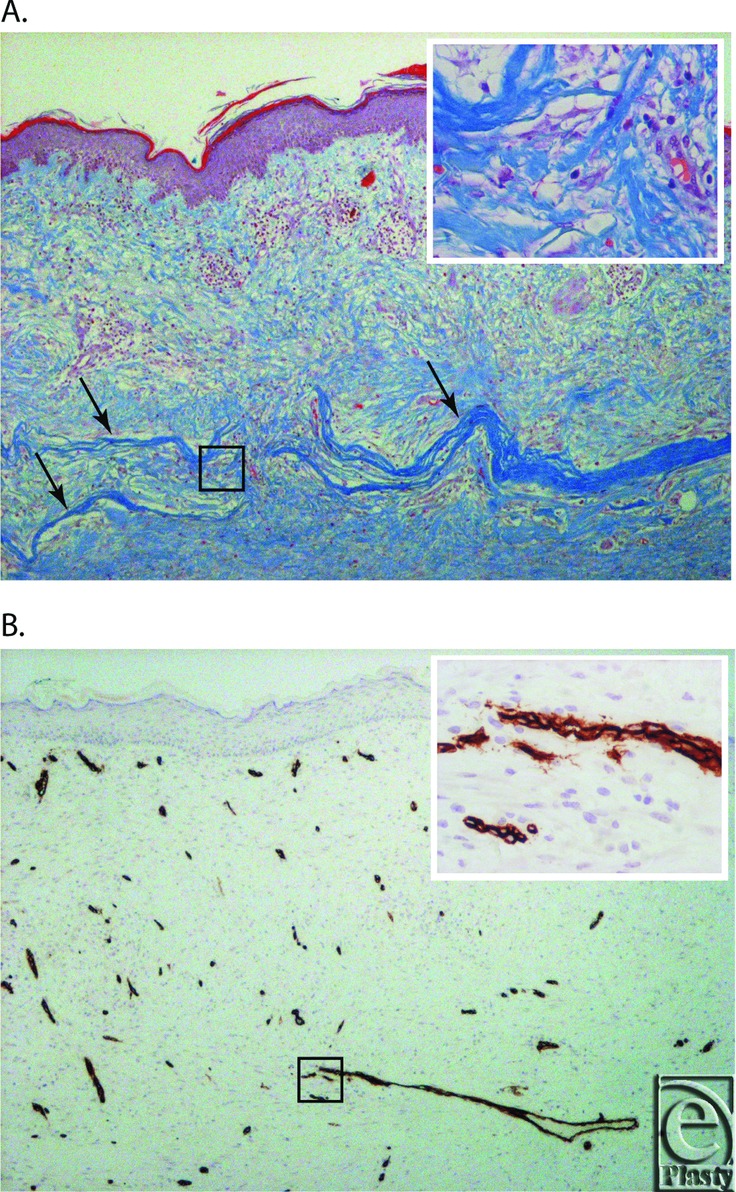
(*a*) Gomori's Trichome stain of the excised graft from B004, 4 weeks postgraft (4× magnification). Arrows indicate the intact fragments of OFM. Insert shows a 40× magnification of the area indicated by the black square. (*b*) CD34 immunohistochemistry of the excised graft from B004, 4 weeks postgraft (4× magnification). Insert shows a 40× magnification of the area indicated by the black square.

Two of the participants (B004 and B005) had the original surgical site further excised 4 weeks postsurgery to gain adequate (>1 mm histological margin) excision of the tumors at the deep margin. The subsequent procedure excised the original graft as well as the margins and underlying periosteum leaving exposed skull. Therefore, the defects were closed with scalp rotation flaps. The excised tissues containing the original graft were fixed, stained, and imaged (Fig 3a). Remnants of the matrix was evident in both B004 and B005 appearing as compact blue collagen fibers that were distinct from collagen of the regenerating dermis. The matrix was evident in the upper sections of the regenerating dermis, immediately beneath the superficial dermis from the STSG. Matrix fragments were infiltrated with fibroblasts and immune cells, including multinuclear giant cells (MNGCs) macrophages and lymphocytes. The immune response in B005 was greater than that in B004, with mononuclear cells and MNGCs associated with the remodelled matrix. Both patients had a well-vascularized dermal layer with dense well-organized collagen bundles and spindle-shaped fibroblasts (Fig 3a). A fully formed keratinized stratified squamous epithelial layer was present and dermal papillae extended into the epithelial layer. An extensive network of blood vessels was present within the regenerating dermis, as evidenced by anti-CD34 immunohistochemistry (Fig 3b).

## DISCUSSION

Scalp reconstruction is especially challenging given the limited blood supply of the underlying calvaria, the relatively thin cutaneous tissue, and the lack of redundant skin. Split-thickness skin grafts take well on the underlying periosteum; however, this leaves an obvious contour defect. Skin flaps and expanders have been traditionally used, but these approaches are complicated by the minimal laxity of the scalp and the complexity of these multistage procedures. As an alternative, collagen-based biomaterials that function as temporary dermal scaffolds have become increasingly useful as part of a single- or 2-stage procedure for surgical reconstruction. These materials allow direct grafting to the underlying calvaria, usually following removal of the outer portion of exposed bone to allow vascularization of the dermal scaffold.^7^^,^^12^^,^^13^ There are a few examples in the literature where dermal scaffolds have been used directly in contact with exposed pericranium to support an STSG,^8^ and to our knowledge this is the first report of a xenogenic dermal scaffold being used in this fashion. The current composite grafting procedure allows for a single-stage procedure to be completed, therefore reducing increased costs associated with multiple procedures and longer term wound management. Results from the 5 participants enrolled in the current study indicate that clinical outcomes from this approach were not compromised, though further controlled studies are warranted.

Previous preclinical studies have shown OFM is remodelled, and importantly the remodelling phenotype resolves with time, with concomitant deposition of new tissues.^10^ This is consistent with the known inflammatory response invoked by decellularized extracellular matrix–based biomaterials, namely remodelling as characterized by an immunomodulatory M2 macrophage phenotype rather an acute inflammation.^14^ The current study provided a rare opportunity to microscopically examine a snapshot of the remodelling of OFM following human implantation, be it with a limited sample size. As has been seen previously in in vivo studies,^10^^,^^15^ the inflammatory response to OFM included the recruitment of a number of immune modulatory cells, including lymphocytes, macrophages, and MNGCs. Long-term resolution of the remodelling inflammatory response in participants was evidenced by the robustness of the regenerated dermis and absence of any wound breakdown.

While the current application of this procedure was in the reconstruction of tissue deficits following tumor resection, there is the potential for this approach to be applied to the treatment of burns and traumatic skin loss. This initial study also suggests OFM as a candidate substrate for autologous cell seeding, whereby suspensions of dermal cells (eg, keratinocytes or fibroblasts) or stem cells (eg, bone marrow or adipose-derived stem cells) are applied to the substrate. This strategy has many similarities to the composite STSG procedure described here, as it relies on rapid vascularization of the underlying dermal scaffold to support the transplanted cells.

## Figures and Tables

**Table 1:** Inclusion and exclusion criteria

Inclusion criteria	Exclusion criteria
>18 years old	Any cutaneous malignancies with metastatic disease
At least 1 nonmelanoma skin cancer without metastatic disease	Diagnosed with malignant melanoma
Malignancies that require full-thickness excision	Systemic malignancy
Postexcision wounds that would normally be reconstructed with a split skin graft	Under suspicion of metastatic disease
	Pregnant or lactating
Compliant	Clinically significant cardiac, pulmonary, renal, hepatic, neurologic, and/or immune dysfunction that may affect wound healing
Competent	
Tumor located on the scalp, neck, or upper limbs	Known allergy to collagen or ovine (sheep) materials; any previous reaction to a collagen product
	Family or personal history of severe allergies (including asthma, hay fever, and atopic dermatitis)
	Allergies to foods, especially meat products
	Unable to remain in study for 6 mo
	Diabetes mellitus
	Declined, unable, or unwilling to make informed consent
	Not fluent in English or Maori—requires interpreter
	Religious or ethical objections to sheep-derived product
	Previous radiotherapy at the defect site
	Immunosuppressant medication (prednisone >5 mg/d or equivalent)

**Table 2:** Summary of participant details and outcomes

Participant	Sex	Age	Tumor location	Age, mo	Type	Area, cm^2^
B001	Male	83	Left vertex scalp	4	SCC	1.5
B002	Male	83	Left anterior scalp	9	BCC	1.2
B003	Male	73	Vertex scalp	8	Previous SCC	16.0
B004	Male	81	Left vertex scalp	2.5	SCC	2.9
B005	Male	61	Left vertex scalp	6	SCC	4.6
